# Considering Genomic Scans for Selection as Coalescent Model Choice

**DOI:** 10.1093/gbe/evaa093

**Published:** 2020-05-12

**Authors:** Rebecca B Harris, Jeffrey D Jensen

**Affiliations:** School of Life Sciences, Arizona State University

**Keywords:** coalescent theory, population genetics, selective sweeps

## Abstract

First inspired by the seminal work of Lewontin and Krakauer (1973. Distribution of gene frequency as a test of the theory of the selective neutrality of polymorphisms. Genetics 74(1):175–195.) and Maynard Smith and Haigh (1974. The hitch-hiking effect of a favourable gene. Genet Res. 23(1):23–35.), genomic scans for positive selection remain a widely utilized tool in modern population genomic analysis. Yet, the relative frequency and genomic impact of selective sweeps have remained a contentious point in the field for decades, largely owing to an inability to accurately identify their presence and quantify their effects—with current methodologies generally being characterized by low true-positive rates and/or high false-positive rates under many realistic demographic models. Most of these approaches are based on Wright–Fisher assumptions and the Kingman coalescent and generally rely on detecting outlier regions which do not conform to these neutral expectations. However, previous theoretical results have demonstrated that selective sweeps are well characterized by an alternative class of model known as the multiple-merger coalescent. Taken together, this suggests the possibility of not simply identifying regions which reject the Kingman, but rather explicitly testing the relative fit of a genomic window to the multiple-merger coalescent. We describe the advantages of such an approach, which owe to the branching structure differentiating selective and neutral models, and demonstrate improved power under certain demographic scenarios relative to a commonly used approach. However, regions of the demographic parameter space continue to exist in which neither this approach nor existing methodologies have sufficient power to detect selective sweeps.

## Introduction

Genomic scans for positively selected loci, sometimes referred to as hitchhiking mapping (Harr et al. 2002), remain as a standard and widely utilized tool in population genomic analyses, with applications and implications ranging from ecological to clinical (Haasl and Payseur 2016; Jensen et al. 2016; Stephan 2019). Although a wide variety of statistical approaches have been proposed for such mapping, they commonly rely on distinctive genomic signatures impacting levels of variation, the site frequency spectrum (SFS), and patterns of linkage disequilibrium (LD) (Maynard Smith and Haigh 1974; and see the reviews of Nielsen 2005; Crisci et al. 2012; Pavlidis and Alachiotis 2017). Beginning with the well-founded assumption that the majority of the genomes of commonly studied organisms are primarily shaped by genetic drift and direct and linked purifying selection effects (Comeron 2017; Jensen et al. 2019), these methods search for outlier genomic regions which may be consistent with positive selection—with a typical genomic scan resulting in a range of localized genomic windows which represent putative sweep candidates based on this criterion.

The summary statistics upon which these scans are based act as a proxy for the underlying genealogical history, with neutral expectations generally being derived from the classic Kingman coalescent framework (Kingman 1982), which in turn is described in the limit of the Wright–Fisher (WF) model (Wright 1931). This framework relies on the expectation that only two lineages coalesce each generation, necessitating the assumption that any individual may only contribute a small number of progeny to the next generation. Yet, a strong selective sweep inherently violates this assumption, as individuals may leave larger numbers of progeny owing to the gain in fitness afforded by the beneficial mutation. In this way, the transit time of a beneficial mutation becomes fast compared with neutral expectations, and this rapid change in frequency between generations generates the frequency spectrum- and LD-based expectations associated with selective sweeps on which existing methodologies are based.

As opposed to simply detecting such outlier loci, however, alternative coalescent models have been studied which are appropriate for directly describing the progeny skew/multiple-branch coalescent events associated with selective sweeps (Durrett and Schweinsberg 2005; and see the reviews of Tellier and Lemaire 2014; Irwin et al. 2016). In this study, we take advantage of this rich but underutilized mathematical population genetic literature from multiple-merger coalescent (MMC) theory, and assess the potential improvement that such models may lend to empirical population genomic scans for positively selected loci. We implement a sliding window MMC versus Kingman approximate Bayesian model choice approach, which specifically assesses whether a given outlier region is indeed well fit by the type of MMC progeny skew associated with a selective sweep. In comparing true-positive rates (TPRs) and false-positive rates (FPRs) of this approach with a commonly used method, we demonstrate via simulation that the inclusion of a consideration of fit to an MMC model has important potential to improve our ability to differentiate nonequilibrium demographic effects (e.g., population size change) from selective effects—a notoriously difficult task, and one that is related to the extreme FPRs often associated with genomic scans (Teshima et al. 2006; Thornton and Jensen 2007; Crisci et al. 2013; Harris et al. 2018). This improvement owes to the fact that although neutral demographic events may alter branch lengths, they do not alter branching structure to create MMC events as with a selective sweep. However, when the internal branches produced by severe bottleneck events are sufficiently short (such that the likelihood of observing a mutation residing on that branch is small), the well-characterized difficulty in distinguishing these models remains (see Barton 1998).

In sum, this work further demonstrates the great utility of developing and incorporating alternative coalescent models in empirical population genomic analyses (Wakeley 2013), as there are a great many evolutionary scenarios for which the Kingman coalescent may not be the optimal choice.

## Materials and Methods

### Simulations

Diploid populations of size *N* were simulated using the forward-in-time simulator SLiM v3 (Haller and Messer 2019). All simulations began with a burn-in of 10 *N* generations of standard, neutral WF conditions.

The “observed” data that were classified consist of long chromosomes (*L *=* *5 Mb; resulting in regions both linked and unlinked to the beneficial mutation under selection models). At the end of the burn-in period, populations were assigned a demographic scenario (equilibrium or population bottleneck; see below section), after which a population either continued under neutral conditions or experienced a deterministic selective sweep from a de novo beneficial mutation. Selective sweeps were simulated with strength 2*Ns* = 100, 250, and 500 (as well as 1,000 under the strongest bottleneck model). These occurred from a de novo beneficial mutation at the center of the 5-Mb region. These observed data were used to assess performance, determining what fraction of neutral scenarios were correctly identified as Kingman, and what fraction of sweep scenarios were correctly identified as MMC.

Such classification was based on the “training data,” which either evolved under: 1) neutral WF conditions (the Kingman coalescent) or 2) short periods of sweepstakes reproduction, in which individuals may contribute a *ψ* proportion of progeny to the next generation (resulting in an MMC when *ψ* becomes large). Model selection was performed in a sliding window (size = 100 kb, step = 50 kb), comparing each window in the observed data to the training data. For each demographic history, we simulated 30,000 instances of the Kingman model and 30,000 instances of the MMC model.

Population parameters were chosen to reflect humans (*N*_e_ = 10e+4, *μ* = 1.2e-8/site/generation,* ρ* = 1e-8/site/generation). To reduce computation time, parameters were scaled by a fixed value of *λ* = 2 (*N*_scaled_*= N*/λ, *μ*_scaled_ = *μ*×*λ*, *ρ*_scaled_ = *ρ*×*λ*).

#### Demographic Models

Data were simulated under a range of demographic models, including equilibrium and bottleneck scenarios of varying intensity. Bottlenecks were modeled in the following way: a population of constant size *N* was reduced to size *βN* at time *t*_b_ (in units of 4 *N* generations) in the past and then recovered instantaneously to the same size at time *t*_r_. Population bottlenecks were simulated for various severities (*β* = 10%, 2%, 0.2%) for 0.005×4 *N* generations. We simulated the beneficial mutation occurring at the time of population recovery, and the population was sampled at the time of fixation. Therefore, the time since the end of the bottleneck is a product of the selective sweep (which is treated as an unknown), as our simulations are dependent upon fixation. To enable an appropriate neutral comparison, neutral (Kingman model) bottleneck simulations were sampled from times ranging from immediately after the bottleneck (*τ*  =  0 generations) to the maximum number of generations necessary for the weakest beneficial mutation considered to reach fixation (ranging from 5,000 to 10,000 generations, depending on the underlying population history). In other words, this allowed a fair comparison between bottleneck simulations with and without selection, as they represent the same distribution of post-bottleneck sampling times.

#### MMC Comparisons

We here utilized one particular type of MMC, the ψ*-*coalescent described by Eldon and Wakeley (2006) (and see Matuszewski et al. 2018). Under this model, the majority of reproductive events in a population of size *N* are of the WF variety yielding, on an average, a single offspring; whereas a single reproduction will result in a multiple-merger event yielding *ψN* offspring. Although there are a variety of potentially relevant MMC models (see review of Tellier and Lemaire 2014), a distinct advantage of the ψ*-*coalescent is the ability to clearly assign a biological interpretation to the model; namely, *ψ* represents the fraction of individuals in the following generation contributed by a single individual in the current generation. The value of *ψ* was chosen from ∼*U*[0.004, 0.08]. The lower bound reflects progeny skews more extreme than the normal variance under Kingman assumptions for populations of the size considered here. The upper bound was chosen based on the distribution of population-wide segregating sites (mean = 14, SD = 10) and the ability to reliably calculate summary statistics for the subsequent ABC analysis; in other words, the MMC model often results in an absence of variation under larger values of *ψ*.

Following Sackman et al. (2019), we utilized a system of subpopulations with migration to achieve the desired sweepstakes reproductive events. In the first generation, a new neutral mutation (*m*_2_) arises in a randomly selected individual. All other generations in the ψ-phase track individuals carrying *m*_2_. Each generation consists of these steps:

1. One individual carrying *m*_2_ is chosen from the population (A) and placed in a separate subpopulation (B) of size *N* = 1. The unidirectional migration rate from B to A is set to *ψ*.
2. One WF generation occurs, with migration from B resulting in the chosen individual contributing *Nψ* of the offspring populating the next generation of A. The remaining *N*(1−*ψ*) offspring come from WF reproductive events.
3. Subpopulation B is removed. The next generation begins.

The ψ phase ends when *m*_2_ reaches fixation and the whole population returns to WF conditions. Summary statistics were sampled at fixation and then at 20 randomly selected time-points.

### Statistical Analyses

Summary statistics (Tajima’s *D*, the number of segregating sites, Fu and Li’s *F*, Fu and Li’s *D*, nucleotide diversity, haplotype diversity, Wall’s *B* and *Q*, Rozas’s ZA and ZZ, and Kelly’s *Zns*) were calculated using the R package *popGenome* (Pfeifer et al. 2014). Prior to analysis, correlation coefficients were estimated and highly correlated parameters (>0.8) were removed; the remaining parameters were then centered and scaled. Consistent with earlier work demonstrating an excess of low- and high-frequency derived variants (Eldon and Wakeley 2006; Matuszewski et al. 2018) and elevated LD (Eldon and Wakeley 2008; Birkner et al. 2013) under MMC models, the statistics capturing these patterns were most informative.

#### Model Selection

For a given demographic scenario, we first determined whether it was possible to differentiate between simulations conducted under the Kingman versus MMC, and their corresponding misclassification rates. We did so by implementing leave-one-out cross-validation (*cv4postpr*) in the R package *abc* (Csillery et al. 2012; and see https://cran.r-project.org/web/packages/abc/abc.pdf). Then, we performed a sliding window analysis of the selective sweep simulations to approximate the posterior probability (*postpr*) that each window belonged to the Kingman versus MMC model. A window was considered “sweep-like” if the probability of the MMC model exceeded the 99% neutral credible interval.

### Method Comparison

We compared the performance of our method to that of the widely used SweeD approach (Pavlidis et al. 2013). This method is a more computationally efficient version of the popular SweepFinder analysis (Nielsen et al. 2005), which itself was an implementation (with several modifications) of the CLRT of Kim and Stephan (2002) for use on genome-scale data. Despite only relying on the SFS, SweeD was chosen as a benchmark as previous studies (Crisci et al. 2013) have well described the Type I and Type II error of SweeD in comparison with SweepFinder, OmegaPlus, iHS, and other common genome scan statistics. For example, Crisci et al. found that these SFS-based packages were generally characterized both by lower TPR and FPR under population bottleneck models, whereas approaches additionally utilizing LD-based predictions had greater power at the expense of inflated false positives.

We analyzed selective sweeps under both equilibrium and bottlenecked population histories using 100 grid points (analogous to our sliding window sizes) using all observed polymorphic sites. To generate a null statistical threshold for calling a region swept or not, we also analyzed neutral data under the same demographic history. To determine the TPRs of MMC model choice versus SweeD, we assessed whether there was a significant test statistic within the region of the selected site.

## Results and Discussion

For each demographic history, two competing model selections were considered: the Kingman and the MMC. Based on the initial theoretical results of Durrett and Schweinsberg (2005), we anticipated that strictly neutral regions would be well fit by the Kingman coalescent, whereas selected regions would be better fit by the MMC. As a first step, leave-one-out cross-validation demonstrated that, for each demographic history here examined, the Kingman and MMC models are indeed discernable (supplementary table 1, Supplementary Material online)—though the MMC model is associated with reduced TPRs and increased FPRs compared with the Kingman.

**Fig. 1.**
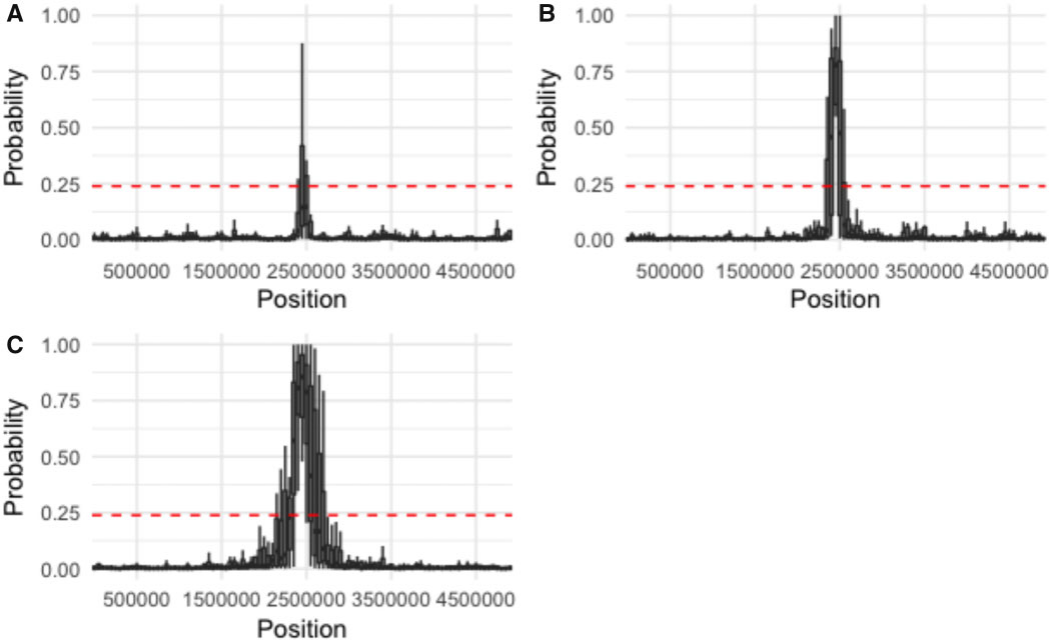
—Probability of accepting the MMC model in a population characterized by equilibrium demography. We simulated 20 replicate deterministic sweeps (a representative replicate is plotted here) occurring from a de novo beneficial mutation at the center of the chromosome (site = 2,500,000) with a strength of (*A*) 2*Ns* = 100, (*B*) 2*Ns* = 250, and (*C*) 2*Ns* = 500. Probabilities were estimated along the 5-Mb chromosome in windows of 100 kb with a step size of 50 kb. The dashed red line indicates the 99% credible interval for accepting the MMC model under neutral conditions. Probability was estimated using the ABC rejection method with a tolerance of 10%. The ψ model included ∼*U*[0.004, 0.8]. As shown, sweep detection is highly accurate under this constant population size model, with the size of the impacted genomic region becoming characterized by underlying MMC trees growing with the strength of selection, as expected.

**Fig. 2.**
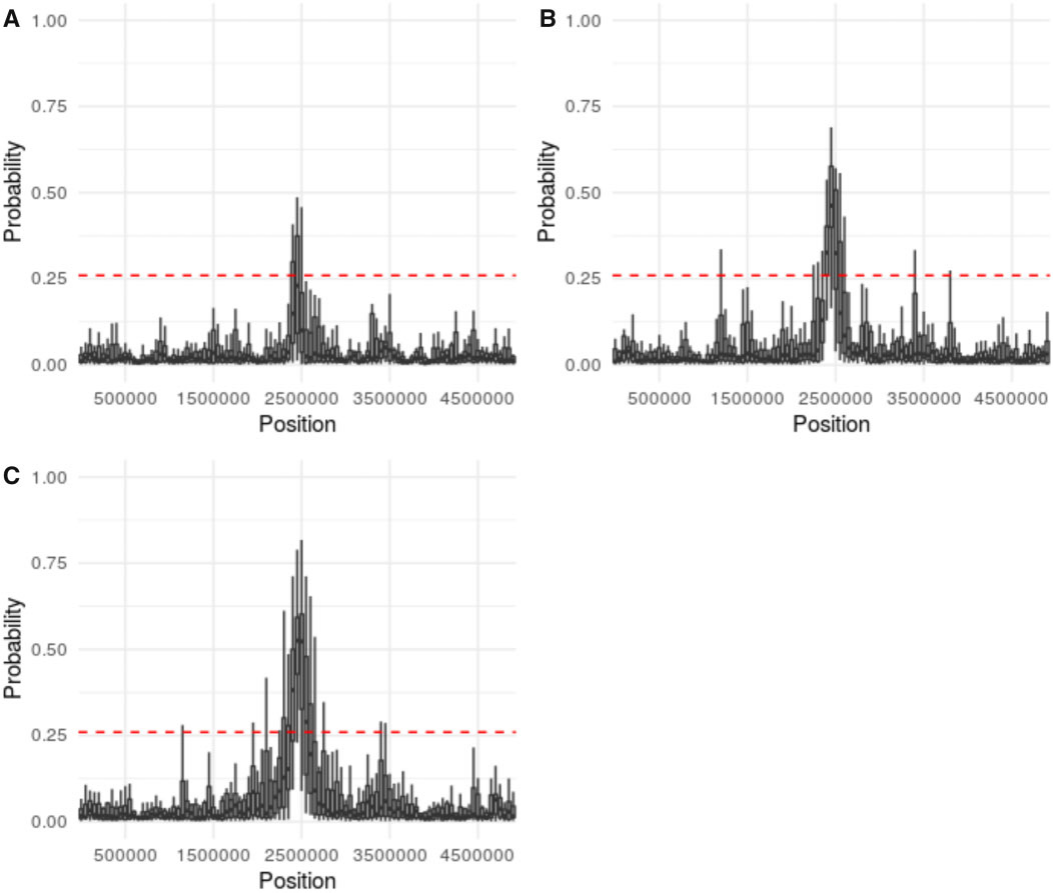
—Probability of accepting the MMC model in a population characterized by a bottlenecked (severity = 10%, duration = 100 generations) demographic history. We simulated 20 replicate sweeps (a representative replicate is plotted here) occurring from a de novo beneficial mutation at the center of the chromosome (site = 2,500,000) with a strength of (*A*) 2*Ns* = 100, (*B*) 2*Ns* = 250, and (*C*) 2*Ns* = 500. Probabilities were estimated along the 5-Mb chromosome in windows of 100 kb with a step size of 50 kb. The dashed red line indicates the 99% credible interval for accepting the MMC model under neutral conditions. Probability was estimated using the ABC rejection method with a tolerance of 10%. The ψ model included ∼*U*[0.004, 0.8]. As shown, performance remains strong as in figure 1, though a proportion of outlier regions now exist in which neutral trees are generated under this bottleneck model which become difficult to distinguish from the MMC trees generated by the selective sweep.

**Fig. 3.**
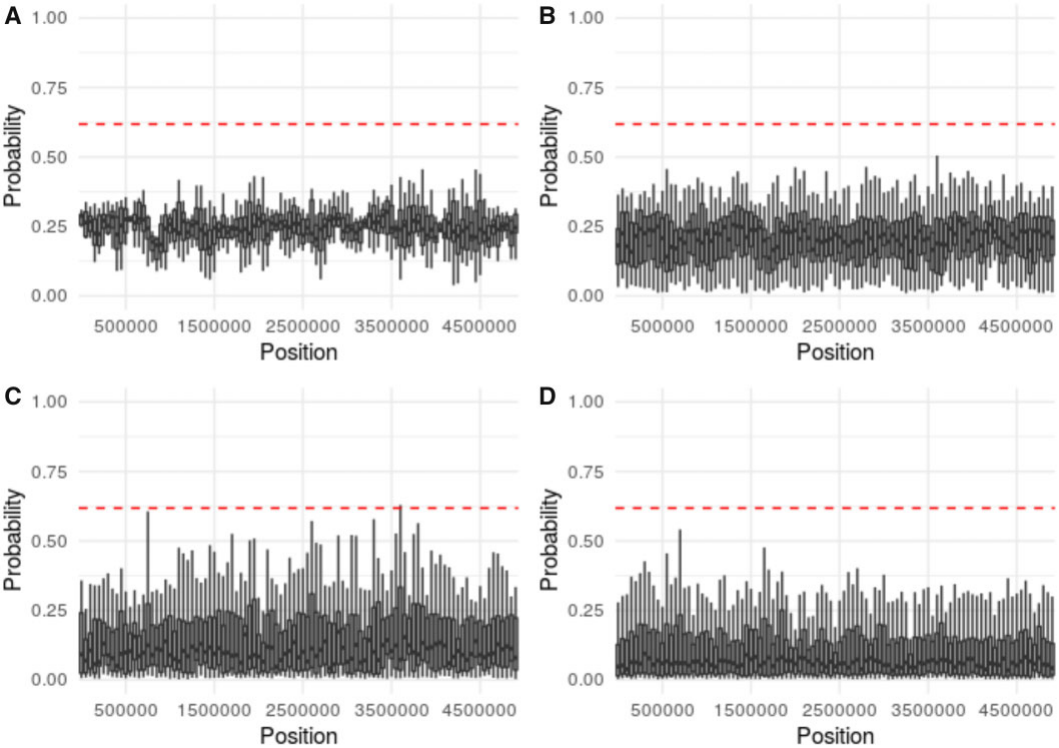
—Probability of accepting the MMC model in a population characterized by a severely bottlenecked (severity = 0.2%, duration = 100 generations) demographic history. We simulated 20 replicate sweeps (a representative replicate is plotted here) occurring from a de novo beneficial mutation at the center of the chromosome (site = 2,500,000) with a strength of (*A*) 2*Ns* = 100, (*B*) 2*Ns* = 250, (*C*) 2*Ns* = 500, and (*D*) 2*Ns* = 1,000. Probabilities were estimated along the 5-Mb chromosome in windows of 100 kb with a step size of 50 kb. The dashed red line indicates the 99% credible interval for accepting the MMC model under neutral conditions. Probability was estimated using the ABC rejection method with a tolerance of 10%. The ψ model included ∼*U*[0.004, 0.8]. As shown, for this extreme bottleneck model, the underlying coalescent trees produced under neutrality relative to those produced by a selective sweep are indistinguishable, consistent with previous work on the topic (Barton 1998; Poh et al. 2014). This performance is thus shared with other existing approaches (table 1). Importantly however, although there is no power to detect selective sweeps under this model, the neutrality threshold for differentiating the MMC versus Kingman models generated under this demographic history is such that false-positive detections are relatively unlikely.

As anticipated based on the bulk of earlier work describing the relative difficulties of distinguishing selective sweeps from bottlenecks of differing severity (Crisci et al. 2013), the threshold for accurately identifying a recently swept region varied by demographic history (supplementary table 2, Supplementary Material online), with more extreme bottlenecks generally resulting in higher thresholds. The performance of the ABC model selection for different demographic histories and strengths of selection may be found in figures 1–3. Under equilibrium demography, positive selection is identifiable even for relatively small selection coefficients (fig. 1). As the bottleneck severity increases, weak positive selection becomes increasingly difficult to detect (fig. 2), until it is eventually not differentiable under extreme population contractions (fig. 3), consistent with previous studies (Poh et al. 2014). 

The above result is simply a feature of the underlying similarity between extreme population bottlenecks and selective sweeps (Barton 1998). As such, no polymorphism-based methods proposed to date have power in this parameter range (Crisci et al. 2013), and existing methodologies claiming to maintain power under such scenarios have been soundly disputed (Harris et al. 2018). Thus, the question under consideration is whether power and FPRs may be improved by the inclusion of MMC model choice in the relatively wide range of demographic parameter space for which it is possible, in principle, to differentiate sweep and bottleneck effects. Encouragingly, in comparison with the most widely used SweeD/Sweepfinder framework, the approach here proposed appears to possess a number of advantages (table 1). By directly assessing the fit of an MMC model, we observed improved power to detect selective sweeps under a variety of bottleneck scenarios. This owes to the fact that although these neutral demographic histories may rescale branch lengths in a manner similar to a selective sweep and reject neutrality using common summary statistics, they do not create multiple-merger events (as does a selective sweep), and thus are not particularly well fit by an MMC model. Conversely, selective sweeps are demonstrated to be poorly fit by the Kingman, but well fit by the MMC. Moreover, as multiple evolutionary processes which result in a localized deficit of variation have been shown to be problematic for variation-based sweep scans—including background selection and heterogeneity in mutation rates (Huber et al. 2016)—it is additionally advantageous that such models are not associated with MMC events. 

**Table 1:** Power Performance of ABC Model Selection versus SweeD

Demography	Selection Coefficient	Coalescent-Model Selection	SweeD
Equilibrium	0.01	0.65	0.1
	0.025	0.89	0.35
	0.05	1.0	0.60
Bottleneck 10%, 0.005×4*N* generations	0.01	0.53	0.09
	0.025	0.87	0.14
	0.05	0.92	0.36
Bottleneck 2%, 0.005×4*N* generations	0.01	0.35	0.025
	0.025	0.85	0.03
	0.05	0.875	0.14
Bottleneck 0.2%, 0.005×4*N* generations	0.01	0	0
	0.025	0	0
	0.05	0	0
	0.1	0	0

Note.—The proportion of sweeps correctly detected within the target window is presented for each demographic history and selection coefficient. As shown, the inclusion of a specific MMC model-fit considerably improves power, allowing for a higher proportion of correctly identified sweeps under multiple bottleneck scenarios. Nonetheless, consistent with the large literature of earlier work on the topic, bottlenecks may become so severe so as to become indistinguishable from selection under either approach.

In sum, our results suggest meaningfully improved power by specifically considering whether a given candidate region is well fit by a multiple-merger coalescent model. However, particularly given elevated FPRs under the MMC relative to Kingman, this model choice approach may be best utilized in concert with standard statistics (such as SweeD), in order to narrow the strongest candidate list. That is, conditional on rejecting Kingman, a second-step model-fit to an MMC appears to be a promising strategy to reduce traditionally high FPRs associated with genome scans. However, as with any sweep-detection methodology, it will be required to quantify the power and FPR of this approach under demographic histories of relevance for any given population-level application of interest.

## Supplementary Material

evaa093_Supplementary_Data
