# Supplementary material for: Considering Genomic Scans for Selection as Coalescent Model Choice
Source: Genome Biol Evol. 2020 May 12;12(6):871–7. doi: 10.1093/gbe/evaa093 (PMC7313662; doi:10.1093/gbe/evaa093)
Supplement: evaa093_Supplementary_Data [file evaa093_supplementary_data.pdf]

Supplement

Supplementary Table 1. Leave-one-out cross validation (*n* = 100, tolerance = 0.01) for model selection with ABC using the rejection method. The MMC model included ~*U*[0.004, 0.8]. As shown, coalescent model choice performs well under equilibrium as well as a variety of increasingly difficult to distinguish non-equilibrium models, though the characterization of the MMC is associated with a reduced TPR and increased FPR relative to the Kingman. Here, the TPR for the Kingman implies acceptance, whereas for the MMC implies rejection - as the true underlying model is a neutral Kingman coalescent.

**Demographic model**	**True Positive Rate**		**False Positive Rate**	
	**Kingman**	**MMC**	**Kingman**	**MMC**
Equilibrium	0.989	0.957	0.011	0.043
Bottleneck 10%, 0.005 * 4*N* generations	0.968	0.918	0.032	0.082
Bottleneck 2%, 0.005 * 4*N* generations	0.913	0.869	0.087	0.131
Bottleneck 0.02%, 0.005 * 4*N* generations	0.918	0.908	0.082	0.092

Supplementary Table 2. Threshold for support of the MMC model, as determined by simulating neutral regions and estimating the probability, implementing an empirical 99% cutoff. The coalescent model threshold thus represents how much more probable an underlying MMC explanation must be in order to reject Kingman, under each demographic model. As shown, this threshold increases with the severity of the population bottleneck, owing to the increasing similarity in the underlying coalescent histories, until a lack of variation begins reducing the threshold for SweeD. For comparison, the likelihood threshold necessary to reject neutrality is given for SweeD.

Demographic model	Coalescent model threshold	SweeD likelihood threshold
Equilibrium	0.239	12.50
Bottleneck 10%, 0.005 * 4*N* generations	0.266	17.49
Bottleneck 2%, 0.005 * 4*N* generations	0.344	27.04
Bottleneck 0.2%, 0.005 * 4*N* generations	0.618	7.09
